# Surface Plasmon Resonance for Biomarker Detection: Advances in Non-invasive Cancer Diagnosis

**DOI:** 10.3389/fchem.2019.00570

**Published:** 2019-08-09

**Authors:** Noemi Bellassai, Roberta D'Agata, Vanessa Jungbluth, Giuseppe Spoto

**Affiliations:** ^1^Dipartimento di Scienze Chimiche, Università degli Studi di Catania, Catania, Italy; ^2^Istituto Nazionale di Biostrutture e Biosistemi, Università degli Studi di Catania, Catania, Italy

**Keywords:** surface plasmon resonance, liquid biopsy, cancer diagnosis, microRNA, exosomes, circulating tumor DNA, circulating tumor cells, protein biomarkers

## Abstract

Biomarker-based cancer analysis has great potential to lead to a better understanding of disease at the molecular level and to improve early diagnosis and monitoring. Unlike conventional tissue biopsy, liquid biopsy allows the detection of a large variety of circulating biomarkers, such as microRNA (miRNA), exosomes, circulating tumor DNA (ctDNA), circulating tumor cells (CTCs), and proteins, in an easily accessible and minimally invasive way. In this review, we describe and evaluate the relevance and applicability of surface plasmon resonance (SPR) and localized SPR (LSPR)-based platforms for the detection of different classes of cancer biomarkers in liquid biopsy samples. Firstly, we critically discuss unsolved problems and issues in capturing and analyzing biomarkers. Secondly, we highlight current challenges which need to be resolved in applying SPR biosensors into clinical practice. Then, we mainly focus on applications of SPR-based platforms that process a patient sample aiming to detect and quantify biomarkers as a minimally invasive liquid biopsy tool for cancer patients appearing over the last 5 years. Finally, we describe the analytical performances of selected SPR biosensor assays and their significant advantages in terms of high sensitivity and specificity as well as accuracy and workflow simplicity.

## Introduction

Clinical diagnosis plays a crucial role in early detection and monitoring of tumor progression. Modern medicine depends on biomolecular information for the diagnosis process, which includes the detection and identification of the pathology, the definition of its burden and stage, and the choice of more appropriate pharmacological treatment. Moreover, the monitoring of the therapeutic response and continuous follow-up of physiopathological conditions, during and after treatments, are critical aspects for improving patients' health and management.

Nowadays, cancer represents a leading cause of death worldwide, accounting for 18.1 million new cases and 9.6 million deaths in 2018, according to the latest report from the International Agency for Research on Cancer (Bray et al., 2018; International Agency for Research on Cancer, 2018). Globally, about one in six deaths is due to cancer, and the total number of people who survive within 5 years of a cancer diagnosis is estimated to be 43.8 million. Currently, cancer employs a massive effect on society and an early, accurate, and sensitive diagnosis, with a description of its molecular landscape, is strictly required in cancer management, as it can lead to effective therapeutic interventions by decreasing the treatment cost and substantially enhancing patient outcome and overall survival (Gorgannezhad et al., 2018).

Standard clinical protocols for the evaluation of tumor profiling are usually based on tissue biopsy, which among several forms of direct tumor biopsy, consists of sampling cells from the human body using special needles or surgery (Crowley et al., 2013). Although this method allows examining the structure and the features of invasive tumor lesions, the tissue biopsy shows disadvantages in terms of functional profiling of oncogenic mutations. For example, a few tumors evolve in some anatomical locations not always accessible for a biopsy and, in many cases, the tissue extraction may augment the risk of metastatic lesions (Robertson and Baxter, 2011). Repeated surgical operations are also needed to follow tumor progression or when the intratumor heterogeneity is not adequately represented by the samples tissue. Tissue biopsy methodology is time-consuming, costly and requires an operating theater for the sample collection. Moreover, even if different metastatic areas could be simultaneously examined, a significant delay could be recorded at the beginning of the pharmacological treatment due to the long time-analysis, compromising the prognosis (Crowley et al., 2013).

**Figure 1:**
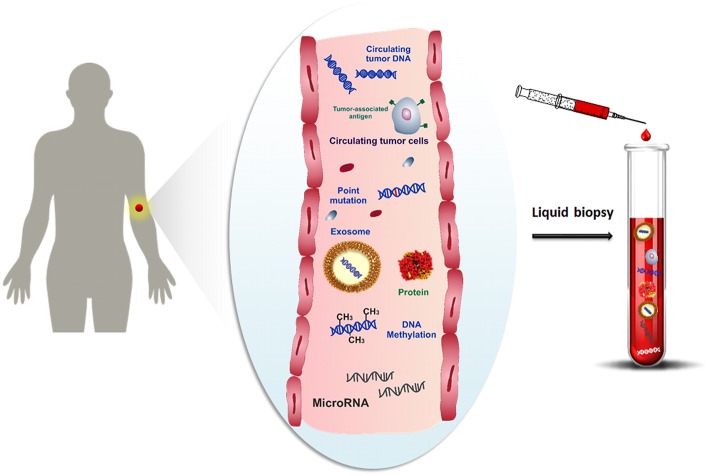
Schematic representation of cancer-related biomolecules such as cells, proteins, nucleic acids and microvesicles circulating into the bloodstream, and collection of these biomarkers by liquid biopsy.

One promising alternative to overcome the limitations of the tissue biopsy is the analysis of cancer cells and any other cancer-related biomolecules (such as nucleic acids, proteins, microvesicles, etc.), combined with their microenvironment, for detecting and monitoring the disease progression at several time intervals by using bodily fluids. Over the last decade, several studies have been focused on the study of molecular biomarkers in tumor diseases by liquid biopsy (Shigeyasu et al., 2017; Lodewijk et al., 2018; Marrugo-Ramírez et al., 2018; Neumann et al., 2018). Liquid biopsy, also known as fluid biopsy, consists of the detection of tumor-derived materials (e.g., circulating tumor cells (CTCs) (Alix-Panabières and Pantel, 2014), circulating tumor nucleic acids (ctNAs, i.e., circulating cell-free tumor DNA, ctDNA) (Thierry et al., 2016; Wan et al., 2017), tumor-derived RNA (predominantly microRNAs) (He et al., 2015), tumor-derived extracellular vesicles (microvesicles, exosomes) (Zhang et al., 2017) and proteins (Borrebaeck, 2017), obtained in a minimally or non-invasive way through the sampling of body fluids, such as blood, plasma, serum, urine, pleural effusion, cerebrospinal fluid and saliva (Mandel and Métais, 1948; Peng et al., 2017) (Figure 1). The evaluation of cancer biomarkers by liquid biopsy might be used for diagnosis, prognosis, determination of cancer predisposition, and predicting response to (targeted) therapy (Alix-Panabières and Pantel, 2016). Through the non-invasive sampling, liquid biopsy becomes more feasible for real-time monitoring of disease progression than tissue biopsy. Moreover, the development of reliable, reproducible, and highly sensitive technologies related to the liquid biopsy makes liquid biopsy an attractive tool for early cancer diagnosis (Palmirotta et al., 2018).

## Biosensors in Clinical Diagnostics

Laboratory diagnosis is usually made on the basis of highly sensitive and specific test results obtained with cell culture methods (histopathology/cytology), enzyme-linked immunosorbent assays (ELISA), next-generation sequencing (NGS), and polymerase chain reaction (PCR)-based platforms which are broadly applicable to different classes of biomolecules and are designed to be highly efficient for processing relatively large numbers of samples. These conventional methods come with laborious, multi-step and time-consuming procedures such as (1) the complex sample protocol, (2) the time-consuming process (days for DNA sequencing, hours for PCR-related methods) (Thierry et al., 2014), (3) relatively large volume input requirements (approximately 1 mL of biofluids) (García-Olmo et al., 2013), (4) the introduction of potential sources of bias owing to sample contamination and PCR errors (Li and Fan, 2012), and (5) relatively high cost per analysis. Additionally, the above technologies require fully equipped laboratories and specialized personnel to perform the analysis, confining the accessibility of these techniques to sophisticated centers, often at the expense of lower speed of analysis.

Microarray technology is offering a highly efficient option for simultaneous identification and determination of a broad range of biomolecules. Microarray substrate is composed of a regularly distributed pattern of DNA sequences or proteins, attached to a solid support, and is able to bind complementary nucleotide sequences and detect mutations or relevant biomarkers in a sample using a label, for example a fluorescence tag. Although this technology started in the 1980s, recent advances in nanomaterials and nanofabrication techniques improved the multiplexing capabilities as well as the detection sensitivity and proposed accurate, rapid and high-throughput screening, already used to investigate and profile the fundamental causes of numerous human diseases and to design new therapeutic drugs (Lěvěque et al., 2013).

Most promising alternative solutions for diagnosis or therapy monitoring of relevant diseases, such as allergy, celiac and diabetes diseases, neurological disorders or cancer, are expected from biosensor devices, which can offer rapid and reliable biomedical analysis (Wang et al., 2017), by employing low sample volumes with minimum pretreatment. Biosensors represent excellent analytical tools for the effective clinical diagnosis as well as for better comprehension of the molecular mechanisms involved in the pathophysiology, by revealing new biomarkers useful for the evaluation of appropriate pharmaceutical treatments (Bellassai and Spoto, 2016). The molecular analysis allows detecting *de novo* mutations, which confer resistance, or mutations not responsive to the clinical treatment, which may be sensitive to alternative targeted therapies. At the same time, the development of this molecular assay enhances the monitoring of cancer patients during specific treatment, as well as the therapeutic resistance of tumor cell clones.

In this perspective, early diagnosis and monitoring of pathological conditions, especially for cancer disease, through molecular biomarker analysis by biosensor platforms, may significantly improve prognosis and survival rates, reducing disease burden and helping social development, opening the door to global healthcare access. Among the different biosensing techniques, plasmonic sensor platforms are able to analyze different classes of biomolecules of clinical interest (Mariani and Minunni, 2014) and, especially, the performances of the SPR technique to monitor label-free interactions and to quantitatively detect biomolecules in real-time with high throughput has established its role in clinical diagnosis. Here, we review advances made over the last 5 years in the SPR and LSPR-based platforms for the detection of different cancer biomarkers in liquid biopsy samples. Firstly, we critically dealt with unsolved issues in capturing and analyzing biomarkers by highlighting the current challenges which need to be resolved in applying SPR biosensors for clinical practice. Then, we mainly focused on recent applications of SPR-based platforms to detect and quantify biomarkers in biofluids directly collected from cancer patients. Finally, we describe the analytical performances of selected SPR bioassays and their significant advantages in terms of high sensitivity and specificity as well as accuracy and workflow simplicity for their future application in clinical practice. The discussed contributions have been thus selected by evaluating the analytical performances in terms of sensitivity and specificity as well as accuracy not only in the buffer but, especially, in biological fluids for future applications in clinical diagnosis.

### SPR and LSPR (Bio)sensors for Liquid Biopsy

Generally, SPR sensing can examine the interactions between biomolecules based on affinity binding analysis, including antibody-antigen (Hearty et al., 2018), ligand-receptor kinetics (Carroll et al., 2016; Teran and Nugent, 2019), enzyme-substrate reaction (Massumi Miyazaki et al., 2017), and epitope mapping (Bhandari et al., 2019). SPR is often used as a complementary method to analyze conformational changes study rather than as a primary technique. This application has been used to observe structural changes in protein-small molecule interactions (Mukherjee et al., 2018), proteins under different environmental conditions (Hoarau et al., 2017) or impacts on apoptosis inducers (Nguyen et al., 2015). Another extension of SPR-based detection application is its use in point mutation detection of unamplified genomic DNA evaluated by using plant, bovine and human genomic DNAs (D'Agata et al., 2010). The limits suffered by SPR for the parallel detection of different probe/target interactions are overcome by SPR imaging (Bocková et al., 2019). The possibility to detect unamplified genomic DNA by using an SPRi-based multiplexed assay has been first shown by detecting the testis-specific protein, Y-encoded (TSPY) gene located in the Y-chromosome of the human genomic DNA (Goodrich et al., 2004). Then, the possibility of detecting sequences of the Y-chromosome in pregnant women was evaluated to identify the gender of fetuses by SPR-based biosensors by analyzing the circulating DNA extracted from the peripheral plasma samples collected from pregnant women at different weeks of gestation (Breveglieri et al., 2016).

Nowadays, SPR has also been employed for the detection of antibodies, drugs, and hormones, among other biomolecular markers. Exhaustive papers report on the working principle of SPR in detail but this feature goes beyond the purpose of this review (Nguyen et al., 2015; Hinman et al., 2018). Taking into account only the set-up of the underlying plasmonic-based detection techniques (Homola, 2008; Couture et al., 2013; Li et al., 2015a), SPR sensing can be combined with different transducer configurations, such as the classical Kretschmann configuration, which includes SPRi (Hinman et al., 2018) as well as nanoparticle-based LSPR (Li et al., 2015b), long-range SPR (Jing et al., 2019), fiber-optic configuration (Gupta and Kant, 2018), and phase sensing (Yesilkoy et al., 2018).

Thanks to the sensitivity, the robustness and versatility of this technique, all of the SPR configurations can be exploited for the molecular analysis of cancer-related biomolecules (Jayanthi et al., 2017; Ferhan et al., 2018). Typically, the median concentration of biomarkers detected in plasma from cancer patients is lower than 20 ng mL^−1^ at an early stage of the disease or higher in metastatic tumors, while several studies reported lower median concentrations in healthy donors (pg mL^−1^ range) (Spindler et al., 2015; Szpechcinski et al., 2015). SPR sensing can detect biomolecules in these concentration ranges where picomolar to nanomolar detection limits are ordinary (Singh, 2016). Moreover, SPR biosensors can perform the analysis in a broad range of biofluids (Masson, 2016), including plasma, serum, urine, whole blood and saliva by highlighting the versatility of these sensing platforms in complex matrices and getting most of the potential clinical samples.

One of the main challenges in nanoplasmonic biosensing is to ensure high sensitivity and specificity for the biomarker detection at the early disease directly in a real sample and without the need of any labeling system to generate the detected signal (label-free). The combination of extremely low concentration of cancer-related biomolecules, especially at the initial stage of disease, with the molecular complexity of biofluids, which profoundly affects the reliability of the signal detection, requires new plasmonic biosensing methods which should achieve stable and robust signal amplification, background signal suppression and non-specific binding prevention needed to enhance the analytic performance. To optimize the cancer biomarker detection in clinical diagnosis, excellent SPR performances have encouraged the study of innovative strategies for the amplification of transducer signals for the target detection at lower levels. For example, the secondary detection by using antibodies or modified nanoparticles is by far the most common signal amplification method implemented to date in the clinical field (Im et al., 2014; Lévêque et al., 2014; Lu et al., 2016a; Aura et al., 2017). In this perspective, nanotechnology offers unique opportunities for creating highly sensitive plasmonic biosensing devices and ultrasensitive bioassays. Signal amplification can be achieved with SPR-active nanostructures, which can employ nanoparticles (D'Agata et al., 2011; Zeng et al., 2014), plasmonic nanostructures of greater refractive index sensitivity (Live et al., 2012) or nanostructures with field depth confined closer to the surface (Couture et al., 2013), leading to larger SPR shifts for a given detection event.

Biointerfaces based on nanomaterials are particularly suitable for the development of improved DNA detection assays (Samanta and Medintz, 2016; Huang et al., 2018). Among metallic nanostructures, gold nanoparticles (AuNPs) have been so far the most useful and extensively exploited for improved DNA detection, thanks to their fascinating electronic and optical properties (D'Agata et al., 2017b). The increase of sensitivity by using AuNPs mainly depends on three factors: (i) an increment of the absolute mass in each binding event, (ii) a rise in the bulk refractive index of the analyte, and (iii) electromagnetic interaction between the localized surface plasmon (LSP) of metallic nanoparticles and SPR of the sensing film. The resonant excitation of LSPs is determined by the size, the shape, and the surrounding dielectric environment of the plasmonic nanostructure. SPR signal amplification by secondary detection is also a powerful method to prevent the background effects and to ensure the quantification of the analyte directly in biofluids. However, the detection by antibodies or nanoparticles increases the costs and the time per analysis, and the complexity of the assay due to a greater number of steps in the procedure. Then, the selective capture and quantification of low numbers of the target molecules with high sensitivity, low background response from biofluids and, possibly, with no secondary amplification are critical aspects for the development of an effective and functional plasmonic biosensor. In light of these requirements, the development of SPR surfaces imposes the optimization of the functionalization process, especially for the selection of specific bio-receptors for the target binding along with the prevention of non-specific adsorption of undesired biomolecules coming from the biofluids. Specifically, for SPR biosensors, non-specific adsorption may cause functional device interference, possibly preventing the detection of biological targets available at low concentrations in complex media. Surfaces may be chemically modified to produce antifouling layers able of decreasing, or ideally suppressing, the fouling effect due to non-specific protein linkage (Thompson et al., 2016) and, at the same time, to keep the activity of the biorecognition elements attached to the surface for the detection of the specific biomarkers. The layer packing density and orientation, its activity and stability during the analysis time and, especially for nanostructured substrates, the selective binding solely onto the active sensing areas are crucial elements for an ideal immobilization of antifouling materials. The thickness of the functional layers should be relatively close to the surface (<100 nm) since the sensing field of nanoplasmonic devices, limited by the decay length (*l*_d_) of the evanescent plasmonic field, rapidly decays into the dielectric medium (Soler et al., 2019).

Many surface chemistries have been applied to minimize the non-specific adsorption of biomolecules to surfaces (Blaszykowski et al., 2012). Common antifouling materials are based on poly(ethylene glycol)/oligo(ethylene glycol) (PEG/OEG)-based materials (Chen et al., 2010; Damodaran and Murthy, 2016), single amino acids, polysaccharides (Liu et al., 2016), zwitterionic compounds such as phosphorylcholine-based derivatives (Chen et al., 2017), betaines (Vaisocherová et al., 2015), mixed-charge polymers (Bellassai et al., 2018), and hydrogels (Shen et al., 2019). However, the low fouling surface chemistry can restrict the binding of the biomolecular receptors onto the surface or decrease their efficiency in capturing the analyte. In addition to the antifouling polymers, blocking reagents could be added to the SPR chip, in the sample or in the running buffer in order to minimize the non-specific adsorption of undesired biomolecules. Otherwise, the use of the microfluidic devices coupled to the SPR instruments requires low sample volume and, therefore, would achieve high interaction of the biological sample with the plasmonic surface while reducing the possibility of the blockage for the bioreceptor binding sites available for the target detection.

Currently, research efforts are directed toward solving the remaining issues for the application of SPR sensing in clinical practices by improving the sensitivity and reliability in contemporary SPR techniques, especially for LSPR platforms. In particular, important issues to be addressed arise from the analysis in the whole complex medium, the evaluation of different types of biological receptors for robust and sensitive detection, and the clinical variability of the biofluids.

LSPR offers many advantages in performing colorimetric detection, which could eventually guide to the naked eye (Basso et al., 2015; Yockell-Lelièvre et al., 2016) or smartphone-based sensing (Liu et al., 2015; Dutta et al., 2016; Wang et al., 2016), and the application of new nanostructures, such as AuNPs in microfluidic-based biosensing, is highly promising for the optimization of the sensitivity in point-of-care devices (Sun et al., 2014; Guo et al., 2015; Giuffrida et al., 2018). LSPR-based biosensors can work in two different approaches: solution-phase or surface-bound sensors. Solution-based biosensors allow simplifying the assay detection, although the stability of colloidal nanoparticles in complex fluids is not guaranteed due to both the formation of a protein corona and the ionic strength of the biofluids. In this direction, the surface functionalization of nanoparticles, the biological scaffolds, the size selection and the dilution of the biological sample can minimize the influence on LSPR sensors of complex media for sensing clinical samples (Aćimović et al., 2014; Singh, 2017). Surface-bound LSPR sensors can resolve the issue related to the stability of the nanoparticle dispersion, since the nanoparticles are bound on a solid substrate, and it shows a more straightforward configuration compared with a multiplex on-chip integrated with microfluidics. For these reasons, the surface-bound LSPR sensors have been employed in a more significant number of clinical studies (Huang et al., 2013; Inci et al., 2013; Joshi et al., 2014a; Chen et al., 2015). In this perspective, we will describe in the following sections some of the most relevant and recent studies of cancer biomarker detection, including miRNA, exosome, DNA, cells, and proteins performed with SPR and LSPR biosensors, selected in the last 5 years, by evaluating the analytical performances of plasmonic biosensors in terms of the robustness, simplicity and reproducibility of the assay for target detection, especially in biological fluids.

### SPR and LSPR Detection of miRNA

miRNA is a single-stranded, non-coding RNA molecule of short size (usually around 18-25 nucleotides length) that plays a crucial role in cellular processes like apoptosis, proliferation, differentiation, invasion, and migration (Kappel and Keller, 2017). The aberrant expression of miRNA has been correlated to many human diseases such as cancer, cardiovascular diseases, and others (Adams et al., 2015; Tao et al., 2015). miRNA exhibits remarkable stability when released into circulation, making it a promising biomarker candidate. However, due to its short length and low abundance and due to high sequence similarity between members of the same family, the detection of miRNA remains complex (Lu et al., 2016b; D'Agata and Spoto, 2019). Established methods to identify and quantify miRNA as microarrays, quantitative reverse transcription PCR (qRT-PCR), electrochemical methods or surface enhanced Raman spectroscopy (SERS)—able to detect highly sensitive structural variations of low concentration analytes through the amplification of electromagnetic fields due to the excitation of localized surface plasmons—do not lead to the required needs to implement miRNA as biomarkers into clinical practice. Good analytical performance (sensitivity, specificity and multiplexing capacity) is often not combined with sufficient usability (high throughput, simplified workflow and cost-effectiveness) (D'Agata and Spoto, 2019). Microarray assays require a complicated procedure, long assay time and expensive reagents (Zhao et al., 2015). qRT-PCR assays need sequence-based amplification prior to detection, labeling steps, the design of suitable primers, and housekeeping genes for normalization of data and optimization of sequence-specific annealing temperature (Peltier and Latham, 2008). SERS is only rarely used for non-SERS active molecules (Ouyang et al., 2016). Therefore, there is a significant need for the development of new and improved technologies to be implemented into clinical workflows. In principle, the working hypothesis of plasmonic sensors relies on detecting changes in the local dielectric environment. miRNA, with only a single nucleotide difference in the sequence, is expected to display nearly identical refractive indices, and thus the change in the local dielectric environment should be identical.

**Figure 2:**
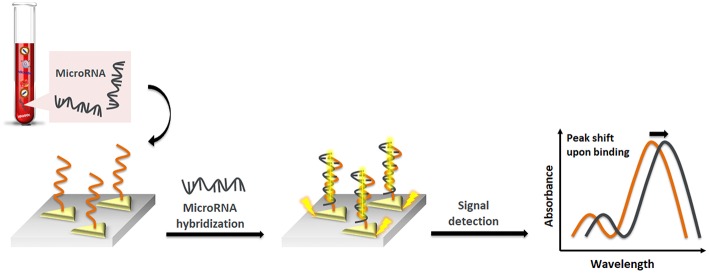
Pictorial description of nanoplasmonic sensors modulating the plasmoelectronic effects at the AuTNP and –S-ssDNA/microRNA interface.

Liyanage et al. (2019) described a new transduction mechanism that involves the delocalization of photoexcited conduction electron wave functions of gold triangular nanoprisms (AuTNPs) in the presence of single-stranded (ss)DNA/miRNA duplexes (Figure 2). The plasmoelectronic effect influences LSPR properties of AuTNPs, thus enhancing the sensing performances. Using this sensor, the level of miR-10b, miR-182, miR143, and miR145 in plasma samples from bladder cancer patients could be detected with high specificity and a limit of detection (LOD) as low as 140 zeptomolar (zM). This ultrasensitive assay does not only have the potential to become a novel liquid biopsy platform but also to detect circulating miRNA in patient plasma for an early-stage, low-volume diagnostic test for various diseases and the analysis of single cancer cells.

Joshi et al. (2014b) recently reported on the first LSPR-based sensing approach in physiological media. They developed a highly specific plasmonic biosensor for ultrasensitive miRNA detection in plasma from pancreatic cancer patients. This assay utilizes gold nanoprisms attached to a glass surface, functionalized with ssDNA (HS-C6-ssDNA) complementary to target miRNA. The direct hybridization of target miRNA to this sensor surface was observed by monitoring the LSPR dipole peak (λ_LSPR_). Using this sensor, the level of miR-21 and miR-10, potential circulating diagnostic and prognostic biomarkers for pancreatic ductal adenocarcinoma (PDAC), could directly be quantified with high accuracy in the sub-femtomolar (fM) range. The LSPR-based measurements showed that the levels of this miRNA are at least 2-fold higher when compared to standard qRT-PCR, where some RNA is lost in sample preparation steps. In this case, any procedure for sample preparation of RNA target sequence (such as modification, amplification or labeling) has been avoided, and all drawbacks related to the current sensing approaches have been overcome. In addition, the sensor could be regenerated without losing sensing efficiency by using DNA-RNA duplex cleaving enzymes. This approach makes the sensor a simple, cost-effective tool for the detection of any miRNA as an early diagnosis of cancer. In their understanding, the high sensitivity of that assay can be explained by the unique LSPR properties of gold nanoprisms with a flat surface, small height (8 nm) and sharp tips. These nanoprisms show strong electromagnetic (EM) field enhancement near the surface and are therefore expected to be very sensitive to changes in the local dielectric environment. Additionally, the transformation of a ssDNA sequence into a double-stranded (ds) structure upon hybridization with target miRNA alters the refractive index significantly due to the high charge density and polarization of DNAs or DNA/miRNA heteroduplexes. A duplex DNA is able to transfer charge over a long range, which changes the electron density around the nanoprisms, influencing the LSPR properties.

However, all of these assays still rely on amplification strategies by PCR, HCR, enzymes, proteins or nanoparticle enhancement. These strategies make the assay time-consuming and prone to problems with non-specific adsorption. Different approaches have been introduced to overcome such limitations, including SERS-based detection. Ding et al. (2015) have recently reported on an SPR biosensor for highly sensitive detection of miRNA based on DNA super-sandwich assemblies and streptavidin signal amplification, which is supposed to overcome these mentioned limitations. In this assay, a thiolate capture hairpin probe complementary to target miRNA is immobilized to the sensor surface. Target miRNA hybridizes with the thiolate probe leading to the conformational change. The capture probe, with hairpin loop, opens up its structure and exposes binding sites for the auxiliary probe AP1, modified with a biotin tag, after the miRNA hybridization. AP1 partially binds another auxiliary probe, AP2, forming a super-sandwich on the sensor surface. A signal enhancement cascade is further triggered by introducing streptavidin to the sensor surface. Streptavidin attaches to the chip surface by binding to the biotin label of AP1 in the super-sandwich. This sensing platform was used to detect miR-21, a potential cancer biomarker with elevated expression levels in various tumor tissues. Direct detection led to a LOD of 470 pM, while the signal enhancement cascade was lowering the LOD down to 9 pM. After calibrating the sensing platform, miR-21 was successfully detected in human breast adenocarcinoma MCF-7 cells, demonstrating a non-compromised analysis in complex components. Furthermore, the assay was shown to highly detect specific miRNA sequences (complementary target miRNA, single-base mismatched target, double-base mismatched target and unspecific miRNAs). In conclusion, this sensing platform allows for simple, rapid (30 min) and enzyme-free detection of miRNA with LOD of 9 pM. Additionally, the assay could be regenerated, allowing the usage of the same chip for at least 20 times.

It is worth citing other platforms which also report on the detection of miRNA from liquid biopsy samples, owing to their versatility which extends well-beyond their use in tumor disease diagnosis by focusing on different disorders. For example, Miao et al. (2016) described a plasmonic colorimetric strategy for visual miRNA detection based on a hybridization chain reaction (HCR) amplification procedure. In their assay, a blocker DNA oligo is immobilized to solid interphase and partially hybridized to an initiator DNA. In the presence of target miRNA, this initiator is released from the blocker, and the target completely hybridizes with blocker DNA. HCR in a gold colloid system with two DNA probes is triggered. ssDNA can interact with AgNPs through its nitrogen-containing bases and acts as a stabilizer of the colloid system, as the aggregation of NPs induced by salts can be prevented. The released initiator DNA, however, binds to that ssDNA on the AgNPs and forms a dsDNA, which does not expose nitrogen bases anymore and therefore can no longer interact with the NPs. The HCR consequently occurs due to the transformation of ssDNA to dsDNA. The stabilization ability of the DNA in the colloid system is changed. This variation is then reflected in the extinction spectrum. Using this calibrated sensing approach, the concentration of miR-29-3p, a biomarker for the H1N1 virus, was detected in real samples. A commercial qRT-PCR kit was used as a control, providing consistent results with the new sensing approach. This colorimetric assay offers a LOD of <1 pM, with the advantage of easy sampling and assay operation and great practical utility. Sguassero et al. (2019) report on a simple and universal enzyme-free approach for the detection of multiple miRNAs using a single nanostructured enhancer of SPRi. They immobilize DNA probes complementary to target miRNA to a solid surface. Upon hybridization of the target, a universal nanoenhancer is used to amplify the signal. This nanoenhancer consists of NPs immobilized with an anti-DNA/RNA antibody that binds to the DNA/RNA duplex on the surface. Using this assay, a LOD of up to 0.5 pM for the simultaneous detection of four different miRNAs related to multiple sclerosis was achieved. Compared to other sensing approaches, this method allows the detection of theoretically unlimited numbers of target miRNA, due to the enhancing properties of AuNPs functionalized with a commercially available antibody recognizing any RNA/DNA duplex.

### SPR and LSPR Detection of Exosomes or Extracellular Vesicles

Exosomes are extracellular nanovesicles (ranging in size from 30 to 150 nm in diameter) actively secreted by eukaryotic cells (Simpson et al., 2009). These extracellular vesicles are broadly present in different body fluids, such as blood (Caby et al., 2005), urine (Duijvesz et al., 2010), saliva (Yang et al., 2014) and cerebrospinal fluid (CSF) (Chen et al., 2013), easily obtained in a minimally-invasive way (An et al., 2015). Exosomes carry several nucleic-acid- and protein-rich contents with molecular information resulting from the cancer cells that reproduce the genetic or signaling modification of the parent tumors, thereby offering great potential for non-invasive cancer diagnostics (Wang et al., 2018). Compared to CTCs and ctDNA, exosomes are abundantly released from tumor cells (≥10^9^ vesicles mL^−1^ in blood Kowal et al., 2014), they show very high structural stability, prolonged circulation time and are capable of protecting from degradation proteins and nucleic acids in the cargo through their lipid bilayer. Encouraging results with clinical relevance in patients with pancreatic or ovarian cancer have been achieved (Melo et al., 2015). However, current conventional techniques for the isolation and quantification of exosomes such as ultracentrifugation, nanoparticle tracking analysis (NTA), Western blot, ELISA and flow cytometry are not convenient, do not lead to a high-purity isolation, require large amounts of sample input and all of these approaches lack specificity (Kibria et al., 2016; Tian et al., 2018).

Much effort has been made to develop new strategies based on microfluidic technologies (Liga et al., 2015) combined with SPR sensors, whose high sensitivity for exosome detection is associated with the fact that exosome sizes perfectly match within their sensing range (Im et al., 2017).

**Figure 3:**
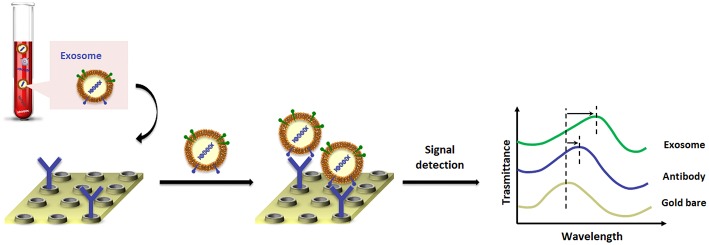
Pictorial description of changes in transmission spectra for the exosome detection by nano-plasmonic exosome (nPLEX) assay.

A landmark paper from Im et al. (2014) reported on nano-plasmonic exosome (nPLEX) technology, in which the sensing is based on transmission SPR through periodic nanohole arrays for the continuous and simultaneous isolation, detection and high-throughput quantification of exosomes. The nPLEX consisted of a series of nanohole arrays with a diameter of 200 nm and a periodicity of 450 nm; each nanohole array has been functionalized with antibodies that recognize exosome surface proteins (Figure 3). The chip design exploited a configuration with parallel fluidic channels modified to allow the capture and the detection of up to 12 different subpopulations of exosomes. In this way, exosomes purified from ovarian cancer cell culture of healthy controls could be readily differentiated from exosomes in ascites samples from ovarian cancer patients. When an exosome binds to one of the nanopore arrays, the target-specific exosome interaction causes a spectral intensity shift in the nanopore optical transmittance which is proportional to the target marker protein levels (with exosomal CD24 and EpCAM as biomarkers). By combining these nanopore chip arrays with a miniaturized imaging set-up, the authors developed the first microfluidic platform with high-level integration and multiplexing capabilities. The chip can be scaled for real-time monitoring of molecular binding measurements (10^5^ independent nanopore arrays), with a sample volume required for each measurement of only 0.3 μL, demonstrating a 97% diagnostic accuracy and drastically improving the lower-limit detection down to ~3,000 exosomes (670 aM sensitivity).

The complex and costly nanostructures fabrication restricts the applicability of nanoplasmonic biosensors comparing with traditional SPR biosensors. So, to avoid any nanostructure fabrication process, Liu et al. (2018) presented a mini-compact SPR biosensor for exosomal protein detection as a user-friendly platform, which may serve as an *in vitro* diagnostic test for cancer diagnosis. However, although the miniaturization of the SPR sensor can extend the applicability to clinical settings, it cannot compete with the high sensitivity of some of the described SPR systems. In any case, by using exosomal epidermal growth factor receptor (EGFR) and programmed death-ligand 1 (PD-L1) as biomarkers, they described the successful lung cancer diagnosis and SPR detection of exosomal EGFR at concentrations as low as 2 × 10^10^ exosomes mL^−1^, by exhibiting better sensitivity than ELISA.

A typical SPRi format combined with antibody microarrays has been exploited to reveal and quantify the exosomes in the central nervous system (CNS), taking advantage of the exosome size matches of the decay length of the evanescent plasmonic field, lowering the sensitivity level and providing the detection of multiple exosome subpopulations of central origin directly in blood (Zhu et al., 2014). In the same manner, by analyzing exosome subpopulations in blood derived from neurons and oligodendrocytes, Picciolini et al. (2018) demonstrated the heterogeneity in exosome populations in terms of phenotypic expression and abundance reporting an SPRi assay.

Rupert et al. (2016) described an SPR-based platform that employs dual wavelengths, which not only detects the presence but also precisely measure the sizes and the concentrations of exosome subpopulations.

By selecting a custom-made adaptation of the SPR platform, a two-step process to isolate and quantify (without multiplexing) the proportion of tumor-derived exosome subpopulation within the bulk population from patient serum has been implemented (Sina et al., 2016). First, exosomes have been captured on a gold surface by using antibodies against ubiquitous exosomal CD9 and CD63 biomarkers; next, a label-free immunoaffinity assay of cancer-specific exosomes expressing HER2 (HER2+ subpopulation) by using anti-HER2 antibody has been performed. Adopting this strategy, exosomes have been satisfyingly isolated from a small cohort of breast cancer patients with roughly 14–35% of the isolated bulk exosome population expressing HER2. The LOD of this approach was 2,070 exosomes μL^−1^.

The plasmon resonance characteristics of AuNPs and the associated considerable EM field enhancement produced by LSPR excitation has made feasible the design of biosensors for the exosome quantification in liquid biopsies (Duraichelvan et al., 2016). For example, AuNPs functionalized by the non-covalent conjugation with a panel of DNA aptamers, which are able to bind the exosome surface proteins with high specificity and affinity, produced a distinct color change as a consequence of AuNP aggregation (from red to blue) following the specific binding between the aptamers and cell-surface proteins (Jiang et al., 2017). The established colorimetric platform allowed to capture and profile the expression levels of numerous exosome proteins isolated from different types of cancer cells in a multiplexed approach, both by visualizing the color change via the naked eye and by measuring the absorption spectrum.

Lastly, an LSPR biosensor (Thakur et al., 2017) based on self-assembly of gold nanoislands (SAM-AuNIs) has also been designed to detect microvesicles human lung cancer cells, neuroblastoma cells blood serum and urine directly. The SAM-AuNIs were synthesized by a deposition/annealing process and covalently embedded into the glass substrate with a LOD as low as 0.194 μg mL^−1^.

Di Noto et al. (2016) directly combined AuNPs with SPR spectroscopy to perform enhanced profiling of exosomes derived from multiple myeloma (MM), gammopathy of undetermined significance, and healthy individuals, aimed at a differential diagnosis. Through the aggregation of AuNPs, they found that exosome production in cancer patients is 4 to 10 times higher than in healthy people, and among the analyzed exosomes only the MM-derived ones have a significant binding affinity for heparin, a structural analog of heparin sulfate proteoglycans known to mediate exosome endocytosis as verified by SPR spectroscopy. In this configuration, a preliminary estimation of the biosensor LOD in the range of 10 pM has been gained.

Unfortunately, the above-described assays do not apply to the detection of individual exosomes. An LSPR imaging (LSPRi) platform, which significantly improves LOD down to the single exosome limit, is described by Raghu et al. (2018). Individual capture events of exosomes derived from a purified breast cancer cell line have been detected by employing a nano-fabricated gold nanopillar array modified with anti-CD63-antibodies in an LSPRi sensor. These antibodies have been projected to target the exosome proteins secreted by MCF7 breast adenocarcinoma cells. Lithographically patterned gold nanosensors have been designed with a diameter of 90 nm to roughly accommodate at most one exosome by matching approximately the width of a single exosome and individually captured in a real-time image. Then, each gold nanosensor has been placed atop a quartz nanopillar, to isolate it, for reducing unwanted background contribution deriving from non-specific adhesion of the nearby substrate. The sensitivity of the LSPRi platform in exosome detection (injecting a solution of 10^5^ exosomes mL^−1^ at sub-femtomolar concentration over anti-CD63 functionalized nanopillars) has been obtained by the increase of nanostructure brightness resulting from the LSPR peak red shift of 2 nm as well as by an overall increase in scattered peak intensity, enabling highly multiplexed detection with single nanopillar resolution (0.36 μm^2^).

### SPR and LSPR Detection of Circulating Tumor Cells and Tumor DNA

Both CTCs and ctDNA provide deeper insights into the understanding of the dynamic fluctuations and characteristics of the tumor disease, contributing to identify and more accurately to profile various types of cancer (Yi et al., 2017). However, their ultralow abundance, the inherent heterogeneity and the high background levels of circulating wild-type DNA make the detection of CTCs and ctDNA even harder. Nucleic acid biomarkers can be conveniently detected through hybridization reactions with a highly specific surface-immobilized probe having a complementary sequence to the target one. Unfortunately, no research reports exist on ctDNA detection based on a simple SPR scheme and, as described before, a certain number of different approaches, such as the plasmonic signal amplification by metallic nanostructures (Sugumaran et al., 2018) for an ultrasensitive DNA detection (Zanoli et al., 2012; D'Agata and Spoto, 2013), have been used to achieve enhanced sensitivities in detecting nucleic acids (Jayanthi et al., 2017).

Remarkably, even if the size of ctDNA is within the sensing penetration depth of LSPR sensors, only a few papers concerning the LSPR detection of ctDNA have been reported. Two main reasons are responsible for that: first, adopting the aggregation-based strategies in solution means that the conjugation of ~100–200 nucleic acid base pairs long ctDNA (Underhill et al., 2016) to the single NP will prevent NPs from approaching each other to get a distance for plasmonic coupling to happen (Jain et al., 2007); second, the mutated ctDNA fragment may be hidden due to the tendency they have to arrange secondary structures (e.g., homodimers, hairpin, and loops), which decrease the efficiency of the target detection process (Sanromán-Iglesias et al., 2017).

In this context, peptide nucleic acid (PNA) (D'Agata et al., 2017a) probes play a key role in the development of highly sensitive biosensors for detecting target nucleic acids at ultra-low concentrations with the capability to identify single-base mutations (D'Agata et al., 2011). The PNA probe is a valuable and advantageous alternative over the DNA probe since, bearing a neutral like-peptide backbone, it does not have any electrostatic influence on the negatively charged phosphate backbone of target DNA, and thereby the hybridization process is not dependent on salt concentrations. Besides, the duplex PNA/DNA with mismatched DNA is more destabilizing than that in DNA/DNA (D'Agata and Spoto, 2012).

Numerous SPR applications based on PNA probes have been reported (D'Agata et al., 2008, 2010; Ieng et al., 2009). Here, we will describe the most relevant plasmonic platforms able to detect ctDNA or CTCs in biofluids for cancer diagnosis by developing innovative and simple nanoplasmonic assays for label-free target detection in blood with excellent selectivity.

Nguyen and Sim (2015) reported a nanoplasmonic LSPR platform for specific and parallel detection of both tumor-specific mutations and methylation of ctDNA. PNA-functionalized AuNPs were used as the probe to recognize and bind mutations at two hotspots—E542K and E545K—of the PIK3CA gene, whereas a specific anti-5-methylcytosine monoclonal antibody (mAb) employed as epigenetic marks of ctDNA of PIK3CA gene.

In an initial step, the binding of ctDNA fragments to PNA probes (PNA-AuNPs-target genes) produced an LSPR peak red shift (around 4.3 nm) after the exposure of 200 fM of point mutated ctDNAs spiked into human serum samples in contrast to the peak shift of 0.1 nm for the normal circulating DNA. Afterwards, the introduction of mAb-functionalized (mAb)-AuNPs, specific for methylated cytosine, caused signal amplification when the DNA methylation was present in the target sequence, thereby improving the sensitivity of the biosensor. At the optimal temperature of 62°C, they observed a further red shift to 11.4 nm due to plasmonic coupling with the PNA-AuNPs, leading to a discrimination of the hot-spot mutations and epigenetic alterations of the ctDNA and to a decreased LOD to 50 fM.

**Figure 4:**
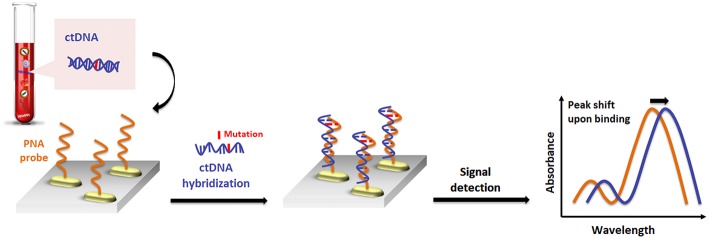
Pictorial description for the sequence-specific detection of circulating tumor DNA (ctDNA) point mutations based on PNA and gold nanorods.

Recently, a similar gold nanorod-based platform (Tadimety et al., 2019) has been proposed for the development of a nanoplasmonic assay for label-free detection of point mutations in the KRAS gene related to pancreatic adenocarcinoma. Gold nanorods were functionalized with PNA sequences capable of recognizing the G12V mutation in the KRAS gene, and the LSPR peak was measured after exposure to synthetic ctDNA sequence both in buffer and spiked in healthy patient serum, establishing a linear detection range below a 125 ng mL^−1^ concentration of ctDNA and LOD of 2 ng mL^−1^ (Figure 4).

By exploiting the effect of the LSPR wavelength, the light intensity and the temperature, a direct plasmon enhanced electrochemical (DPEE) approach has been presented for ultrasensitive and label-free detection of CTCs in blood with excellent selectivity (Wang et al., 2019). Briefly, the glassy carbon electrode was modified with gold nanostars (AuNSs) functionalized with an aptamer capable to selectively capture CTCs spiked in human serum and blood samples, by making the sensor able to discriminate the CTCs from normal cells conveniently. Due to an efficient electron transfer by light irradiation, AuNSs can significantly increase the current response to the electrocatalysis of the electroactive probe (ascorbic acid in this case). Upon the captured cells on the AuNSs surface, the electron transport efficiency from AuNSs to the external circuit is modified, resulting in a reduction of the photocurrent, whose value is proportional to the CTCs concentrations. The dynamic range is 5~1 × 10^5^ cells mL^−1^ and LOD is found to be 5 cells mL^−1^.

### SPR and LSPR Detection of Protein Biomarkers

Circulating proteins represent further promising biomarkers for early cancer diagnosis (Rusling et al., 2010), whose expression, abundance, localization, and biochemical modifications are greatly influenced by any pathological process (Füzéry et al., 2013). In the tumor microenvironment, cancer biomarkers come to the bloodstream through a diffusion process. Even though significant data indicates that thousands of proteins are differentially expressed in human cancers, only a limited number of those are actually checked as biomarkers in clinical practice for cancer diagnosis and monitoring (Polanski and Anderson, 2007).

Immunoassays and affine techniques such as lateral flow immunoassay and immunohistochemistry are consolidated methodologies for protein detection and quantification (Wild, 2013). They are routinely based on a sandwich format providing the necessary selectivity for detection, with a structure made of a highly-specific immobilized antibody and a protein target that is bounded in another portion to a second antibody. This latter, in the ELISA, is combined with a colorimetric enzymatic substrate.

So, with this point of view, we selected the most clinically relevant protein biomarkers already implemented in clinical practice and the use of SPR for their quantification in a real biological sample at lower concentration, by applying nanoparticle-enhanced SPR-based sandwich detection as a potential assay in early cancer diagnosis.

On the basis of their fascinating physical, chemical and electrical properties, AuNPs have been also widely used in optical immunosensors (Kumar et al., 2015). For example, Li et al. (2015a) reported streptavidin-modified AuNPs for SPR detection of carcinoembryonic antigen (CEA) in human serum. In order to improve sensitivity, selectivity and detect CEA at low concentrations, a commercial dextran-coated surface was employed to immobilize the recognition antibody, and after the capture of CEA, a biotinylated secondary antibody was introduced for SPR signal amplification via biotin-streptavidin interaction. As demonstrated in this report, the nanoparticle-enhanced SPR-based sandwich detection method allowed for the determination of CEA at a clinically relevant concentration (below 1.0 ng mL^−1^), obtaining an SPR detection more sensitive than a direct assay approach without AuNPs.

An optimized sandwich nanoparticle-enhanced SPR method has also been used for the detection of various proteins, as well as the ErbB2 receptor tyrosine kinase 2 in human serum and raw cancer lysates. The ErbB2 assay described by Eletxigerra et al. (2016) led to a discrete LOD of 180 pg mL^−1^ when carried out in 50% human serum samples. The detected concentration was several times lower than the clinical cut-off, clearly demonstrating the potential advantage in early tumor detection. Moreover, raw cancer cell lysate samples from model breast cancer cell lines have been analyzed with satisfying accuracy.

**Figure 5:**
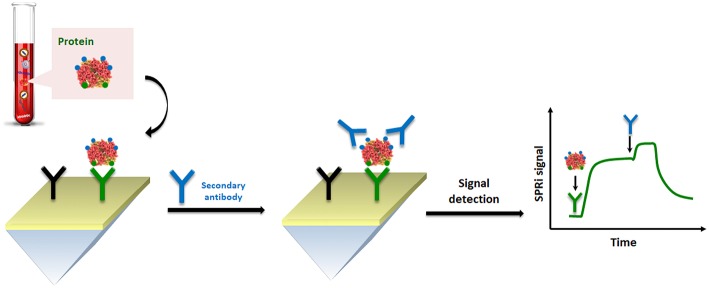
Pictorial description of SPRi sensing of HER-specific exosome (protein) in breast cancer cell lysates.

By considering the nature of cell lysate in comparison to blood-based matrices, such as plasma and serum, spreading ionic liquids (Benedetto and Ballone, 2018) on the surface of the SPR sensor could significantly improve the detection. Indeed, the problem of fouling becomes particularly significant when the SPR analysis is performed using cell lysates which release a large variety of other macromolecules, including lipids and nucleic acids. In this context, Aubé et al. (2017) reported on the use of an ionic liquid self-assembled monolayer which exhibits superior antifouling performance for analysis in a crude cell lysate compared to standard antifouling surface chemistries designed for plasma and serum. Their results successfully demonstrate that ionic liquid may be used to accomplish an efficient HER2 biomarker detection in breast cancer cell lysates (Figure 5).

By using the microcontact imprinting technique, the capture of target prostate-specific antigen (PSA) on the SPR sensor surface without functionalizing the sensing surface with a bioreceptor has been obtained by Ertürk et al. (2016). PSA-imprinted gold coated SPR chips have been designed in the presence of PSA-modified glass cover, used as protein stamp, through a UV-induced photopolymerization process with methacrylic acid (MAA) as a functional monomer and ethylene glycol dimethacrylate as a crosslinker agent. The detection has been firstly performed in buffered standard solutions, measured through a calibration curve, and validated by parallel ELISA determination obtaining an excellent LOD of 91 pg mL^−1^ (18 × 10^−14^ M) within a concentration range of 0.1–50.0 ng mL^−1^. The described SPR sensor also showed high selectivity in the presence of other potential interferences, such as human serum albumin and lysozyme, and reusability of about fifty times. Furthermore, by testing 10 serum samples from prostate cancer patients, the obtained results have been compared with those acquired using the commercial ELISA, and a considerable degree of concurrence between the two methods has been found.

## Conclusions

In the present review, we focused on some of the latest SPR and LSPR applications for the detection of different kinds of circulating tumor markers as critical components of liquid biopsy for cancer diagnosis, and how the advances in the SPR technology have improved their detection. Analysis of microRNA, exosomes, CTCs, ctDNA and proteins, which are sampled non-invasively by a simple blood draw, provides an appealing alternative to the traditional tumor biopsy methods for early, non-invasive diagnosis and real-time monitoring of patient conditions. The application of a liquid biopsy procedure has already improved the detection and monitoring of some relevant oncogenic mutations in cancer patients (Finotti et al., 2018). Although the tissue biopsies play a crucial role for confirming and getting a proper understanding of cancer mechanisms, the intra-tumor heterogeneity and the pool of markers released from not biopsied tissues or from hidden micrometastases could lead to different results of tumor profiling among the real samples. Then, precise diagnosis and management should aspire to simultaneously analyze all of these biomarkers in a single liquid biopsy sample.

The discussed SPR and LSPR-based platforms enhance the performances in terms of low sample consumption, multiplexing analysis, specificity and sensitivity. Moreover, they are shown to be able to detect biomarkers on a simple workflow effectively, and particularly for exosomes analysis, they can reach a detection limit down to single molecule by isolating a subpopulation of disease-specific exosomes using a single platform. This capability mainly derives from the small sensing volume and penetration depth, which is on the same length scale as many clinically relevant biomarkers.

Despite the remarkable applicability and performances of SPR-based sensors for cancer biomarker detection in the last few years (Table 1), several issues in the field of SPR sensing, however, need to be overcome for making the SPR technology fully suitable for clinical applications. Indeed, most of the reviewed SPR-based platforms are still at proof-of-concept levels, and their precision and interferences, in comparison with another assay such as ELISA, have been validated against synthetic or spiked-in samples that have been diluted with buffer to minimize non-specific adsorption. Even if many antifouling strategies exist for SPR-based biosensors, few studies have shown to improve the sensitivity of target detection in the undiluted complex media.

**Table 1:** Performances of plasmonic platforms for detection of circulating biomarkers in biofluids.

**Tumor type**	**Biomarker**	**Biological specimen**	**Detection limit**	**References**
Bladder cancer	miRNA (miR-10b, miR-182, miR143, and miR145)	Plasma	140 zM	Liyanage et al., 2019
Pancreatic ductal adenocarcinoma (PDAC)	miRNA (miR-21 and miR-10)	Plasma	23–35 fM (miR-21)	Joshi et al., 2014b
Ovarian cancer	Exosome	Human ovarian cancer cell line culture	3000 exosomes (670 aM)	Im et al., 2014
Lung cancer	Exosomal proteins: epidermal growth factor receptor (EGFR) and programmed death-ligand 1 (PD-L1)	Non-small cell lung cancer (NSCLC) cells/normal human bronchial epithelial cell cultures	2 × 10^10^ exosomes mL^−1^	Liu et al., 2018
Metastasis and neurodegenerative diseases	Exosome derived from central nervous system (CNS)	Blood/Plasma	1 μg mL^−1^	Picciolini et al., 2018
Breast cancer	HER2-specific exosome	Serum	2070 exosomes μL^−1^	Sina et al., 2016
Lung cancer	Microvescicles	Human lung cancer cells, neuroblastoma cells/blood serum, and urine	0.194 μg mL^−1^	Thakur et al., 2017
Multiple Myeloma (MM)	Exosome	Serum	10 pM	Di Noto et al., 2016
Breast cancer	Exosome and exosomal proteins: endosome-specific tetraspanins (CD9 and CD63)	Breast adenocarcinoma cell line	1 single exosome	Raghu et al., 2018
Breast, colon, brain, liver, stomach, and lung cancers	Mutations (E542K and E545K) and methylation of ctDNA of PIK3CA gene	Spiked human serum	50 fM	Nguyen and Sim, 2015
Pancreatic adenocarcinoma	G12V mutation in the KRAS gene	Buffer/spiked human serum	2 ng mL^−1^	Tadimety et al., 2019
Acute Lymphoblastic Leukemia (ALL), breast cancer	CCRF-CEM (CCL-119, T cell line)	Spiked human serum/blood	5 cells mL^−1^	Wang et al., 2019
	MCF-7 cells		10 cells mL^−1^	Wang et al., 2019
Gastrointestinal, breast and lung cancers	Carcinoembryonic antigen (CEA)	Spiked human serum	1.0 ng mL ^−1^	Li et al., 2015b
Breast cancer	ErbB2 receptor tyrosine kinase 2	Spiked human serum/raw cancer lysates	180 pg mL^−1^	Eletxigerra et al., 2016
Breast cancer	HER2	Breast cancer cell lysates	–	Aubé et al., 2017
Prostate cancer	Prostate-specific antigen (PSA)	Serum	91 pg mL^−1^	Ertürk et al., 2016

In parallel, it has been reported on several successful approaches to increase the SPR/LSPR signal for the biomarker detection at very low concentrations. Sandwich strategies and/or an aggregate of nanoparticles to improve the detection limit when revealing very low concentrated biomarkers have been adopted. Also, PNA probes have received considerable attention for the detection of nucleic acid biomarkers among the receptor type immobilized on the sensing surface, whereas the antibodies remain the most frequently used for detecting circulating proteins.

We believe that future developments in integrating of SPR technology in micro/nanofluidic devices are expected to provide cost-competitive, robust and sensitive strategies aimed at personalized medical applications. Other important aspects to consider for future research contributions in this area are related to the potential competitiveness of plasmonic platforms with digital PCR and NGS, the golden standard technologies for ctDNA detection. With this aim, future research efforts should focus on improving the specificity of the plasmonic detection of mutated DNA that circulates in the peripheral blood of cancer patients with an allele frequency often ranging between 0.1 and 0.01%. The throughput of the analysis is also an essential issue to face given performances of NGS platforms and the continuous identification of new mutations with clinical relevance for cancer treatment. The need to adequately address the aspects mentioned above may be a partial justification for the current development of plasmonic platforms for biomarker detection at the proof-of-concept level.

## Data Availability

The raw data supporting the conclusions of this manuscript will be made available by the authors, without undue reservation, to any qualified researcher.

## Author Contributions

NB, RD'A, VJ, and GS designed this study. NB, RD'A, and VJ wrote the manuscript. GS revised the manuscript.

### Conflict of Interest Statement

The authors declare that the research was conducted in the absence of any commercial or financial relationships that could be construed as a potential conflict of interest.
